# Reassessing Mandatory Folic Acid Fortification for Neural Tube Defect Prevention: Evidence, Uncertainty, and Policy Implications

**DOI:** 10.3390/nu18111758

**Published:** 2026-05-30

**Authors:** Cara J. Westmark

**Affiliations:** 1 Department of Neurology, University of Wisconsin, Madison, WI 53706, USA; westmark@wisc.edu; Tel.: +1-608-262-9730; 2 Molecular Environmental Toxicology Center, University of Wisconsin, Madison, WI 53706, USA

**Keywords:** folate, folic acid, neural tube defect, personalized medicine, public health, supplements

## Abstract

Background/Objectives: Folate is a water-soluble B vitamin that is essential for DNA synthesis, cell division and proper growth and development, particularly during pregnancy to prevent neural tube defects (NTDs). A large, fully randomized clinical trial (RCT) from the United Kingdom in 1991 (the Medical Research Council (MRC) Vitamin Study), where participants had a prior NTD-affected pregnancy, demonstrated a 72% reduction in NTD recurrence in the folic acid treatment group. Based on this data and the high rate of unplanned pregnancies, about 90 countries fortify cereal grains with folic acid with the goal to prevent NTDs during pregnancy. This critical narrative review and policy perspective addresses the difference between folate and folic acid and between supplementation and food fortification, critically evaluates the data in three recent publications supporting mandatory fortification of food products with folic acid, and presents the case for a more personalized medicine approach to the prevention of NTDs. Methods: Relevant literature was identified through PubMed searches using the keywords “fortification”, “folic acid”, and “systematic review” or by Googe Scholar Alerts. Three studies were identified based on relevance to the topic and publication dates between January and February of 2026. Results: There is a disregard in published studies, which use pre-fortification groups as controls, for the confounding issue of changing socioeconomic factors over time. Improved socioeconomic conditions are associated with subsequent decreases in NTD prevalence regardless of fortification. Conclusions: The efficacy of folic acid supplementation for recurrent NTDs is supported by evidence-based literature, but evidence in favor of mandatory food fortification to prevent NTDs is limited. Food fortification is widely debated, raises numerous ethical issues, and has broad implications for human health.

## 1. Introduction

In 1998, the United States implemented mandatory fortification of cereal grains with folic acid to prevent neural tube defects (NTDs) during pregnancy [1]. The mandate has been debated with proponents citing the need for national fortification due to the high prevalence of NTDs and unplanned pregnancies in conjunction with the need for early exposure for disease prevention and opponents voicing concerns about adverse health effects and the ethics of non-consent, inability to opt out, unevenly distributed risks, and lack of transparency regarding potential harm. The objectives of this critical narrative review with policy perspective are to address the differences between folate and folic acid and between supplementation and food fortification, as well as to critically evaluate the data in several recent publications supporting mandatory fortification of food products with folic acid and to present the case for a more personalized medicine approach for the prevention of NTDs. The circumstances that inspired conception of this study arose from our prior findings of lack of efficacy of folic acid food fortification in reducing NTDs at the population level and a strong linear relationship between reduced NTDs and better SES.

## 2. Folate Versus Folic Acid

**Figure 1:**
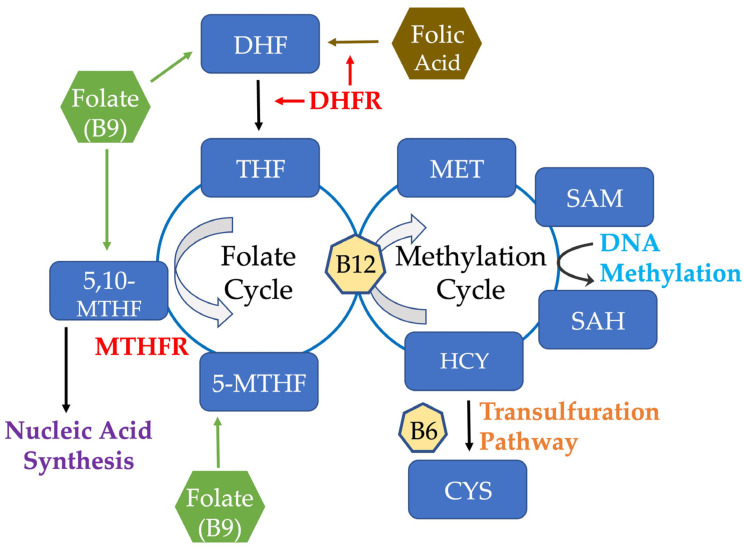
Diagram of one-carbon metabolism.

Folate is a water-soluble B vitamin (B9) found in leafy greens, legumes, citrus fruits and whole grains. It is vital for DNA and RNA synthesis, cell division and growth, red blood cell (RBC) formation and homocysteine metabolism. Folate is especially critical during early pregnancy where it helps prevent NTDs like spina bifida and anencephaly. Folate refers to many related compounds, including folic acid, dihydrofolate (DHF), tetrahydrofolate (THF), 5-methyltetrahydrofolate (5-MTHF), and 5,10-methylenetetrahydrofolate (5,10-MTHF). The principal naturally occurring folates are in the THF form and polyglutamated form. Folate requires enzymatic conversion in the gut to become 5-MTHF, which enters portal circulation and is immediately biologically active without hepatic reduction. MTHF reductase (MTHFR) is the final enzyme in a multistep pathway that converts folate to the active metabolite 5-MTHF, which is the form of folate transported across the intestinal mucosa into cells and across the blood–brain barrier where it functions as a co-substrate in single-carbon transfers for the synthesis of nucleic acids and metabolism of amino acids (Figure 1) [2]. An example is the conversion of homocysteine (Hcy) to methionine (Met) in the synthesis of S-adenyl-methionine (SAM), which becomes S-adenosylhomocysteine (SAH) after donation of a methyl group for DNA methylation reactions. When B12 is deficient, the conversion of Hcy to Met is inhibited, and folate is trapped as 5-MTHF, which cannot serve as a substrate for thymidine synthesis.

The synthetic folic acid used to fortify foods and found in most supplements is the fully oxidized mono-glutamate form of folate, which must be converted in the liver by dihydrofolate reductase (DHFR) to DHF followed by THF and finally 5-MTHF. Human DHFR activity is low and highly variable, making this process slow and inefficient in some people and leading to unmetabolized folic acid (UMFA) in the bloodstream. Dietary folate equivalents (DFEs) are used to correct for the fact that folic acid is more readily absorbed than natural folate. One microgram (mcg) of DFE is equivalent to 1 mcg of natural food folate and 0.6 mcg of folic acid consumed with food or 0.5 mcg of folic acid consumed on an empty stomach. Folic acid is used in supplements and food fortification because it is more stable than folate. Folate stability is affected by leaching, heat dehydration and oxidation. For example, water-soluble vitamins like B9 dissolve into cooking water with spinach and broccoli losing half of their folate upon boiling and more dense vegetables like potatoes having no significant loss. Steam cooking preserves folate levels. High cooking temperatures break down folate molecules and exposure to air during cooking can degrade folate. Although less stable, folate is more bioavailable than folic acid [3]. This could be particularly important for persons with *MTHFR* gene variants that impair folic acid metabolism. Due to increased absorption and stability, consumption of high doses of folic acid can lead to UMFA in the bloodstream.

Folates also include 5-formyl-THF (folinic acid) and 10-formyl-THF. Folinic acid (leucovorin) is a stable THF that serves as an intracellular pool of reduced folates and is clinically used to reduce the toxicity of methotrexate (an antifolate) used for chemotherapy. It is found naturally in small amounts in food. It is rapidly metabolized in the body to 5-MTHF without the requirement of initial reduction by DHFR. Conversely, 10-formyl-THF is a short-lived folate and does not accumulate; it serves as an immediate formyl donor and direct substrate for purine synthesis. Folic acid differs biologically from naturally occurring reduced folates including 5-MTHF, folinic acid and 10-formyl-THF in that it requires initial reduction by DHFR before entering the active folate pool. The enzymatic conversion by DHFR is a limiting factor in folate metabolism. The relatively slow process in humans can result in circulating UMFA under conditions of high intake. Reduced folates bypass DHFR. Genetic differences such as MTHFR variants can reduce the conversion of folates to the active form 5-MTHF and are associated with altered folate status and biological effects [4]. These differences in structure and function of folate derivatives underscore the importance of distinguishing synthetic folic acid from reduced folate forms.

In addition to multiple forms of folate with different structures and functions, there are numerous folate transport proteins that affect intracellular pools of folate. Proteins that transport folate include reduced folate carrier (RFC), proton-coupled folate transporter (PCFT), folate receptors (FRs) and mitochondrial folate transporter (MFT). RFC functions in systemic folate uptake in most tissues; PCFT is involved in intestinal and central nervous system (CNS) uptake in the gut and choroid plexus; FRs function in high-affinity endocytosis in placenta, brain and immune cells; and MFT is involved in mitochondrial import. FR-α (a.k.a. folate receptor 1 (FOLR1)) and PCFT are the primary transporters supplying folate to the cerebrospinal fluid (CSF). Deficiencies in the expression and/or activity of these transporter proteins can cause folate deficiency such as that which occurs in cerebral folate deficiency syndrome (CFDS), which is a neurological condition with low 5-MTHF levels in the CSF despite normal blood folate levels [5]. In children, autoantibodies to FR-α are the most frequently identified cause of blocked folate entry into the CNS. Overall, these mechanisms in conjunction with genetic variability underlie differential individual responses to folate and folic acid. In the case of CFDS, the treatment of choice is folinic acid because it bypasses FR-α-mediated transport and increases CSF folate levels. Folic acid is not recommended because it worsens receptor blockade.

## 3. Neural Tube Defects

NTDs are a heterogeneous group of structural birth defects arising from genetic and environmental factors that adversely affect the structure and function of the central nervous system [6]. NTDs occur early in pregnancy, typically 3–4 weeks post-fertilization (approximately 5–6 weeks of gestational age). Anencephaly occurs when the neural tube fails to close at the cranial (rostral) end, leading to absence of major portions of the brain, skull and scalp. It is fatal with no cure or treatment. It is estimated that 1 in every 4762 babies is born with anencephaly in the United States. Encephalocele occurs when a baby is born with a sac-like protrusion of brain tissue emerging from an opening in the skull. These cranial NTDs with skull defects are classified based on the tissue that herniates through the skull opening, i.e., meningocele (meninges) and meningoencephalocele (meninges and brain tissue). Surgery can treat encephalocele to place the protruding brain material back into the skull and close the opening in the skull. It is estimated that about 1 in every 9091 babies is born with encephalocele in the United States. Spina bifida is failure of the neural tube to close at the caudal (lower) end. Spinal NTDs are classified as spina bifida occulta (vertebral defect covered by skin), meningocele (herniation of meninges with fluid-filled sac), and myelomeningocele (herniation of both meninges and spinal cord with neural tissue exposed or covered by a thin membrane). Sometimes surgery can be performed prior to birth to close the spine in myelomeningocele. Many babies born with spina bifida get hydrocephalus, i.e., water on the brain, and require a shunt to drain the fluid. Spina bifida occurs with 1 in every 2875 births in the United States; the highest rates are found in Hispanic women. NTDs are etiologically heterogeneous and folic acid alone is insufficient for prevention as evidenced by persistence of a large proportion of cases after supplementation and/or fortification.

## 4. Public Health Policy

NTDs are among the most common serious congenital anomalies. In response to the United Kingdom Medical Research Council (MRC) study [7], there was concern that women of childbearing age were not consuming enough folate and were at risk of NTD-affected pregnancies. Voluntary supplementation was not perceived as effective. Approximately half of pregnancies are unplanned, and the neural tube closes early in embryonic development. To promote adequate levels of folate prior to becoming pregnant, the United States implemented mandatory food fortification of cereal grains with folic acid in 1998 [1]. Cereal grains were required to be fortified with 0.43–1.4 mg folic acid per pound. The theory was that fortifying a food staple such as white flour with folic acid would result in adequate folate in the target population without a change in dietary pattern. Currently, about 90 countries fortify cereal grains with folic acid. Proponents of mandatory national fortification of food products with folic acid consider the policy medically and financially effective because it utilizes existing food supply chains, requires minimal behavior change, and spreads the cost across the entire population with high coverage and lifetime exposure, thus targeting women of reproductive age before they become pregnant and during gestation. Numerous global and government organizations support mandatory fortification of food products with folic acid (Table 1) [1,8,9,10,11,12,13,14,15,16,17,18,19,20,21].

Opponents of mandatory food fortification with folic acid raise the issue of adverse health effects, particularly for vulnerable populations. While supplementation with folic acid to prevent recurrent NTD pregnancies is documented, fortification is not the same as supplementation and folic acid is not the same as folate. The health benefits of vitamin B9/folate are well-documented; however, folic acid intake from fortification exceeded predictions, with potential health risks of exceeding the upper tolerable limit (UL) in relation to cancer, insulin resistance, adiposity, masking vitamin B12, interaction with antiepileptic and chemotherapy drugs, U-shaped response curves for nervous system function, and slowly changing the genetic make-up of the population, which have been reviewed elsewhere [22,23,24,25,26,27,28,29]. Opponents contend that a personalized approach is required for optimal nutrition and that current fortification policy may be contributing to the substantial economic healthcare burden of debilitating disorders like cancer, autism, and Alzheimer’s disease, which are associated with folate status and hypothesized to be affected by exceeding the UL of folic acid.

## 5. The Evidence

The framework for evidence appraisal distinguishes between evidence for supplementation, evidence for fortification, biological plausibility, safety concerns, and political/ethical considerations.

### 5.1. NTD Prevalence Pre- and Post-Fortification

How good is the evidence-based literature (EBL) regarding food fortification with folic acid? When the United States implemented mandatory fortification of cereal grains with folic acid, policymakers predicted a 70% decrease in NTDs. NTD prevalence during the pre-fortification period of 1995–1996 averaged 7.3 per 10,000 for the White/non-Hispanic population in the Centers for Disease Control and Prevention (CDC) Birth Defects and Developmental Disabilities study [30]. Another predominantly White/non-Hispanic population of 4783 subjects in the National Health and Nutrition Examination Study from 2007–2012, over a decade later and after fortification, likewise exhibited an NTD prevalence of 7.3 per 10,000 live births (range 5.5–9.4 per 10,000 live births) [31]. The current prevalence of NTDs in the United States estimated by the March of Dimes is 3000 births per year [32], which equates to 8.3 per 10,000 for 3.6 million annual births. Regarding spina bifida, a subset of NTDs, the CDC currently reports that spina bifida occurs in 1 in every 2875 births, or 3.5 per 10,000, in the United States per year. Prior to fortification of cereal grains with folic acid in the United States, the CDC analyzed data from 16 states for the incidence of spina bifida at birth from 1983–1990 and found 4.6 cases per 10,000 births with the annual rate declining from a peak of 5.9 cases per 10,000 births in 1984 to 3.2 cases per 10,000 births in 1990 [33]. These data indicate lower or equivalent NTDs and spina bifida pre- compared to post-fortification.

### 5.2. Seminal Studies Used to Support Fortification

Food fortification policy for folic acid in the United States was primarily based on three studies [7,34,35]. First, the United Kingdom MRC Vitamin Study tested the effect of folic acid supplementation in reducing recurrent NTD pregnancies [7]. In a randomized, double-blind prevention trial of 1817 women who were at high risk of having a pregnancy with an NTD because of a previous affected pregnancy and assigned to one of four groups (folic acid, other vitamins, both, neither), the MRC reported a 72% protective effect in recurrent NTD pregnancies with 4 mg folic acid. However, the MRC raised two issues questioning the view of folic acid deficiency as a major cause of NTDs because the United Kingdom had one of the highest rates of NTDs in the world but was unlikely to be unusually deficient in folic acid, and because studies have not shown a significant difference in the serum folic acid concentration of women with affected and unaffected pregnancies. Still, the authors concluded that folic acid supplementation can be recommended for all women who have had a previously affected pregnancy, and public health measures should be taken to ensure that all women of childbearing age receive adequate dietary folic acid.

Second, the Czeizel Hungarian study was a cohort controlled trial of peri-conceptional multivitamin supplementation containing 0.8 mg folic acid on the first occurrence of NTD in women in Hungary with matching to a non-supplemented control for each supplemented woman [34]. A total of 3056 informative offspring (i.e., an informative pregnancy is where the presence or absence of an NTD is ascertainable) were evaluated per cohort with the finding of one (supplemented) versus nine (non-supplemented) NTDs, respectively. The authors conclude there was about 90% efficacy of a peri-conceptional multivitamin supplementation containing folic acid in reducing NTDs. There was no recurrence of NTDs in the supplemented women (*N* = 41) with a prior family history of NTDs.

The Werler study was a multicenter, case–control study in tertiary and birth hospitals in metropolitan areas of Boston, MA; Philadelphia, PA; and Toronto, Ontario from 1988–1991, which compared the use of multivitamins containing folic acid between mothers of cases (*N* = 436 with NTDs) and controls (*N* = 2615 other major malformations). Mothers taking supplements were *N* = 34 and non-supplementing controls were *N* = 339. The authors conclude that peri-conceptual intake of 0.4 mg folic acid reduces the risk of occurrent NTDs by approximately 60% [35]. They note that recall may have been biased among women who knew the hypothesis.

Of note with all three of these studies, the baseline NTD prevalences are very high at 412 per 10,000 in the MRC study; 90 per 10,000 in the Czeizel study; and 1500 per 10,000 in the Werler study. The MRC study population was strictly recurrent NTD pregnancies, which are likely disproportionately affected by genetic, epigenetic, metabolic and/or immune factors rather than folate deficiency alone. The Czeizel study was the only first-time occurrence NTD investigation and tested folic acid in the presence of other micronutrients and not folic acid alone; of note, Hungary has vastly different economic conditions than the United States. The Werler study controls had other major malformations, such as chromosomal anomalies (14%), ventricular septal defects (14%), renal defects (9%), transposition of great vessels (7%), hypospadias (7%), limb reduction defects (7%) and craniosynostosis (5%).

While proponents of fortification cite substantial studies claiming decreased prevalence of NTDs after the implementation of national fortification policies in the United States, Canada, Costa Rica, Chile and South Africa [36,37,38,39,40,41], changing economic conditions spanning pre- and post-fortification periods confound the results and a strong argument can be made that studies showing efficacy do not include appropriate control groups nor consider changing SES. The annual numbers of NTD births were declining prior to mandatory fortification [33,42,43,44,45]. Improved SES is associated with subsequent decreases in NTD prevalence regardless of fortification [46,47].

### 5.3. The Cochrane Library and Folic Acid Fortification

The Cochrane Library is the global leader in systematic reviews and medical evidence and their 95-page evaluation of the efficacy of folic acid fortification of wheat and maize flour on health outcomes in the overall population concluded that there is only one non-RCT that supports the contention that fortification of wheat flour may reduce the risk of NTDs (spina bifida and encephalocele but not anencephaly) [48]. None of the reviewed studies reported the occurrence of adverse effects. Overall, Cochrane found “very low certainty” regarding the efficacy of folic acid fortification in reducing NTDs.

### 5.4. Knowledge Gaps

During the timeframe of pre- to post-fortification of cereal grain with folic acid in the United States, the prevalence of autism, Alzheimer’s disease, obesity and other serious disorders has risen dramatically with current prevalence of autism at 1 in 31 children aged 8 years, Alzheimer’s disease at 1 in 9 Americans aged 65 and older, and obesity at 40% of adults. A critical review of the EBL and prospective monitoring are required to address the hypothesis that folic acid fortification is contributing to these epidemics. Additional gaps in knowledge include specific effects in underrepresented populations and in response to specific genetic variants.

### 5.5. Methods

A PubMed search with the terms “folic acid” AND “fortification” returns over 1800 papers with over 80 systematic reviews. Herein, this narrative review critically evaluates current evidence on the topic of food fortification with an emphasis on folic acid and NTD outcomes. Relevant literature was identified through PubMed searches using the keywords “fortification”, “folic acid”, and “systematic review” or by Google Scholar Alerts. Three systematic reviews were identified based on relevance to the topic, publication dates in January and February of 2026, and author support of mandatory fortification of food products with folic acid. Findings were evaluated to highlight areas of controversy and gaps in the literature. As a narrative review, this work does not aim to be exhaustive but rather to illustrate deficiencies in the EBL regarding support for mandatory food fortification with folic acid. This is not a systematic review or meta-analysis.

### 5.6. Cochrane Response

First, Cochrane Response published a systematic review in collaboration with the Food Fortification Initiative (FFI), Hubert Department of Global Health at Emory University, and USAID Advancing Food Fortification Opportunities to Reinforce Diets, the Department of Hygiene and Epidemiology at the University of Ioannina, Greece, and TechnoServe in Arlington, VA promoting the cost effectiveness of food fortification in reducing global malnutrition [49]. They concluded that their comprehensive systematic review covering a broad scope of economic evaluations finds that food fortification programs are likely cost-effective in most contexts. They claim their findings may assist with evidence-informed decision making for global health policy. The review is based on the premise that the efficacy of food fortification has been well-established, for which they cite an entry in the Cochrane Database of Systematic Reviews by Das and colleagues and a systematic review and meta-analysis by Keats and colleagues [50,51]. Below we evaluate the strength of the evidence in these secondary citations to support health effects associated with fortification of food products with folic acid.

Regarding the Das and colleagues Cochrane review from 2019 [50], their selection criteria included RCTs, cluster-RCTs, quasi-randomized trials, controlled before–after studies and interrupted time series studies assessing the impact of food fortification with multiple micronutrients including folic acid. Primary outcomes included anemia, micronutrient deficiencies, anthropometric measures, morbidity, all-cause mortality and cause-specific mortality. Secondary outcomes included potential adverse outcomes, serum concentration of specific micronutrients, serum hemoglobin levels and neurodevelopmental and cognitive outcomes. Studies included high-, mid- and low-income countries. The main results were all labeled low-quality evidence. There was no information on possible side effects. The authors state they could not perform various subgroup analyses to identify whether fortification is more effective in different population groups, food vehicles, dosages, durations of intervention and geographical regions due to the limited number of studies in each subgroup. None of the included studies reported morbidity, all-cause or cause-specific mortality, or NTDs. The authors’ conclusions were, “The evidence from this review suggests that MMN [multiple micronutrients] fortification when compared to placebo/no intervention may reduce anaemia, iron deficiency anaemia and micronutrient deficiencies (iron, vitamin A, vitamin B2 and vitamin B6). We are uncertain of the effect of MMN fortification on anthropometric measures (HAZ/LAZ, WAZ and WHZ/WLZ). There are no data to suggest possible adverse effects of MMN fortification, and we could not draw reliable conclusions from various subgroup analyses due to a limited number of studies in each subgroup. We remain cautious about the level of commercial funding in this field, and the possibility that this may be associated with higher effect estimates, although subgroup analysis in this review did not demonstrate any impact of commercial funding. These findings are subject to study limitations, imprecision, high heterogeneity and small sample sizes, and we rated most of the evidence low to very low quality. and hence no concrete conclusions could be drawn from the findings of this review”.

Regarding the Keats systematic review and meta-analysis, most of the analyzed publications included NTDs and were conducted in Central and South America. The quality (four moderate, two low) of the studies and citations are provided in their supplementary materials [51], which indicate that all studies under analysis compared odds ratios pre- and post-large-scale food fortification. As previously reported and described below, this is confounded due to changing SES conditions in this part of the globe during the timeframe of fortification implementation.

In summary, the Cochrane Response systematic review promoting the cost effectiveness of food fortification in reducing global malnutrition relied on low-quality and/or confounded evidence in secondary citations to support health effects associated with fortification of food products with folic acid.

### 5.7. Socioeconomic Factors Are Confounding

Murphy and Westmark conducted a population study assessing the effect of folic acid fortification of cereal grains on NTD risk through analysis of the FFI dataset [46]. The FFI monitored 236 countries with data available for 194. Inclusion criteria were all countries with available data on both folic acid fortification and NTD prevalence (186 countries). Eight countries were excluded that were listed as fortifying with folic acid at 0 ppm. Countries with and without national fortification were compared. There was no association between national folic acid fortification and decreased prevalence of NTDs. The average prevalence of NTDs per 10,000 births in countries not fortifying any cereal grains with folic acid was 13.32 ± 5.50 (N = 116 countries), and the average prevalence of NTDs in countries with at least one cereal grain fortified with folic acid was 13.30 ± 6.13 (*N* = 70). Interestingly, NTD prevalence was significantly reduced in response to increased SES. Stratification of the FFI NTD data based on national economic indicators (gross domestic product spent on healthcare, education, social protection) produced a linear relationship between improved SES and reduced prevalence of NTDs (R^2^ = 0.85) with the average prevalence of NTDs in quintile 1 (highest SES) of 10.97 ± 4.83 versus 16.11 ± 6.47 in quintile 5 (lowest SES; *p* = 0.0003) (Table 2).

These data suggest that improved SES contributes to reduced prevalence (>30%) of NTDs and folic acid fortification does not. The 25% increase in NTDs in quintile 3 with fortification compared to non-fortification was statistically significant, *p* = 0.03. The other quintiles were not statistically different as a function of fortification. It remains to be determined if improved NTD outcomes as a function of SES are due to peri-conception folic acid supplementation. There are numerous limitations to this type of observational data including that it is based on retrospective data on folic acid fortification and NTD prevalence; the level of folic acid is declarative; only a single data point is analyzed for fortification levels and NTD prevalence from the year 2013; and lack of data regarding confounding issues such as voluntary fortification, national recommendations for pre-conception and first trimester folic acid intake, and implementation timing of mandatory folic acid programs. However, the advantage of this study is a control group of countries not exposed to mandatory fortification during the same period as the treatment countries.

To begin to address confounding issues, the findings were confirmed by analyzing 33 countries with fortification program coverage of at least 91% where linear regression indicated a very weak correlation between NTD prevalence and folic acid consumption [52]. Considering that there has been no controlled prospective monitoring of health outcomes after implementation of mandatory fortification of folic acid with non-exposed subjects in the same country and under similar SES conditions, this study design constitutes a justifiable strategy to discern health effects in response to fortification. Of note, in low-resource settings such as sub-Saharan Africa, NTDs are likely underestimated due to limited surveillance systems, underreporting, and reduced access to diagnosis. Most data may come from single hospitals versus national registries. Stillbirths, neonatal deaths and home births are frequently underreported. Pregnancy terminations further decrease NTD rates. Reduced access to pre-natal ultrasound and post-natal diagnostic testing reduces NTD identification. Thus, comparison of NTD prevalence between regions needs to be made with caution.

### 5.8. White Paper by National Academy of Medical Sciences

Second, Sharma and colleagues published a white paper on the prevention of NTDs in India authored by the National Academy of Medical Sciences (NAMS) Task Force, which commissioned a blueprint for the Government of India’s Ministry of Health and Family Welfare to eliminate folate and B12 deficiency [53]. They cite two national systematic reviews from India with prevalence of NTDs of 4.1–4.5 babies born with NTDs per 1000 births (41–45 NTDs per 10,000), which is about 6-fold higher than the United States and among the highest in the world. Sharma and colleagues state food fortification with folic acid is highly effective and safe, citing Field and Stover [54]. While Field and Stover conclude that, “there are no established risks for adverse consequences resulting from existing mandatory folic acid fortification programs”, they acknowledge that, “the functional and health consequences, if any, of UMFA are not established” and “the mechanism whereby folic acid prevents NTDs is unknown, limiting the ability to predict unintended clinical consequences”. Richmond and colleagues showed that maternal folic acid supplement use is associated with alterations in DNA methylation in offspring that persisted for 47 years after in utero exposure [55]. Methylation changes were found in genes implicated in embryonic development and cellular proliferation (*Pax8*) as well as immune response (*HLA-DPB1*, *VTRNA2-1*). The Richmond study population was from the Aberdeen Folic Acid Supplementation Trial (AFAST), which was an RCT of two different doses of folic acid (0.2 or 5 mg per day versus placebo) during pregnancy performed in the late 1960s.

Sharma and colleagues recommend fortification of food vehicles with folate and vitamin B12 in conjunction with an education campaign in India for NTD prevention. While they do not make a case for mandatory vitamin fortification of food, they suggest that, after clinical trials for efficacy in widely separated states in India, the government should make various food vehicles available and allow women to choose. They base their decision on the principle that education and experience help sway decision making and lead to increased adoption. They recognize that the prevalence of NTDs is higher in SES-disadvantaged areas of India as they discuss a population-based door-to-door study from Balrampur District, in Uttar Pradesh, which is the least developed region of India and has an incidence of NTDs at 65.7 to 82.1 cases per 10,000 live births. They acknowledge that, prior to widespread release of one or more vitamin-fortified foods, clinical research should be conducted to document efficacy.

Issues requiring more consideration by Sharma and colleagues include: (1) they use the terms folate and folic acid interchangeably; (2) they propose monitoring fortification efficacy by monitoring optimum serum folate and vitamin B12 level to reduce the risk of a folate-responsive NTD in preparatory work; and (3) they rely heavily on expert opinion and not high-level EBL in supporting their recommendations. Structural and biological differences between folate and folic acid have already been discussed. Monitoring blood levels of folate prior to but not after fortification of food products does not take into consideration how individuals respond to fortification. In addition, the proposal to test serum instead of RBC folate levels is problematic. Serum folate levels are dependent on recent dietary intake whereas RBC levels reflect folate status over the lifespan of the cell (~120 days). In persons with the *MTHFR 677T/T* genotype, folate intake is not correlated with plasma levels [56]. The reliance on expert opinion from government agencies that food fortification of wheat flour with folic acid is a safe, cost-effective, large-scale public health initiative to prevent NTDs is not supported by clinical trials or prospective monitoring. The argument could be made that improving the overall SES status of the poorest regions of India would bring their NTD rates in line with other parts of the country.

### 5.9. Systematic Review Covering Pre- and Post-Fortification Periods

Third, Moges and colleagues published a systematic review and meta-analysis on the effectiveness of mandatory folic acid fortification compared with pre-fortification periods in reducing NTDs [57]. They cite 14 studies encompassing 33 million people to support their conclusion that fortification was associated with a 44% decrease in the risk of NTDs. Regarding the 14 studies, they state four were from Canada, two from South America, two from Chile and one each from Jordan, Costa Rica, Peru, the United States, Australia and Brazil (Table 3) [30,45,58,59,60,61,62,63,64,65,66,67,68,69]. Of note, four of the studies contained data for Chile with an overlapping study population for the three working with the Latin American Collaborative Study of Congenital Malformations (ECLAMC) network [58,65,68].

A problem with this meta-analysis is the control groups for the 14 studies. The treatment groups were fortification of staple foods with folic acid, and the control groups were pre-folic acid fortification. The authors claim a 44% reduced risk of NTDs (risk ratio 0.56) but do not consider the documented decline of NTD prevalence over time prior to fortification nor the changing SES of the countries under study. NTD rates were decreasing in the United States, Canada and Australia prior to fortification [33,42,43,44,45]. For studies that graphed NTDs over a large timeframe, there was high variability between years; for example, in the Lopez-Camillo and colleagues study, 1982 is like 2001 with many peaks in between [65]. Without longer follow-up, it is difficult to discern if post-fortification reductions in NTDs are natural troughs in the data. The gross domestic product for all countries in the meta-analysis increased significantly between pre- and post-fortification periods.

Specific examples of economic conditions that likely confound the findings by Moges and colleagues include a large shift from a state-led to open economy in Costa Rica in the 1990s with trade liberalization and a transition from exporting coffee, bananas and sugar to medical devices, electronics, manufactured goods, processed agriculture products and tourism services. Intel built a $300 million semiconductor plant that began operations in 1998. Chile started to rebuild its political system in 1990 from a military to a democratic-based government, resulting in higher expenditure on social programs. Nova Scotia fishing communities were affected by the 1992 Northern Cod Moratorium that collapsed the cod fishing industry, i.e., the backbone of the economy. The moratorium was accompanied by reduced federal funding for healthcare, social services and regional development. The gross domestic product (GDP) did not turn positive until the late 1990s as new industries expanded, including shellfish fisheries, tourism and later offshore oil.

Moges and colleagues contrast their findings with Murphy and Westmark’s population-level comparison of folic acid food fortification efficacy [46], which was reviewed above. They attribute the difference in the results to the impact of geographical and temporal variations in supplementation practices and fortification practices, as well as that effectiveness of fortification may be dependent on population adherence to supplementation recommendations, pregnancy planning and the distribution of fortified foods. Moges et al. conclude that there is a preponderance of evidence to support the overall benefit of mandatory folic acid fortification in preventing NTDs; however, the potentially confounding issues they state would have affected both studies included an array of geographical regions. Murphy and Westmark study included all geographical regions for the year 2013 and Moges and colleagues subgrouped their studies based on geographical region to accommodate heterogeneity between studies. The average NTD rate per 10,000 births post-fortification for the 12 Moges studies providing total NTD prevalences, and averaging data from the same country for equal weighting, was 8.0 (SD 2.6). Analysis of Murphy and Westmark NTD data for only the countries (Argentina, Australia, Brazil, Canada, Chile, Costa Rica, Jordan, Peru, United States) included in Moges indicates an average NTD prevalence of 9.0 (SD 3.5) per 10,000 births with fortification. A Student *t*-test indicates the results are not statistically different, *p* = 0.49. What differs between the studies is the control group. Moges et al. used pre-fortification as the control group and did not consider significant changes in national SES factors. Murphy and Westmark conducted a population-level study comparing countries with/without fortification. They found a strong linear response of reduced NTDs with better SES.

In summary, Moges et al. conclude that there is a preponderance of evidence to support the overall benefit of mandatory folic acid fortification in preventing NTDs. They recognize regional factors such as varying fortification levels, dietary proclivities, compliance with supplementation guidelines, and genetic differences, but they do not account for a major confounding issue of changing SES conditions in their analysis. Malnutrition in general contributes to increased NTDs by disrupting energy availability, one-carbon metabolism, redox balance and epigenetic regulation during neural tube development. Improving SES conditions would be expected to provide access to healthy nutrient- and folate-rich foods and improved healthcare that could underlie reduced NTDs during the timeframe spanning pre- and post-fortification.

### 5.10. Cochrane Reviews

Two final studies to examine are Cochrane reviews on folic acid supplementation and fortification. Supplementation evidence demonstrating folic acid reduced recurrent NTDs was the basis of the current fortification programs. A Cochrane review reported a 69% reduction in NTD risk associated with folic acid supplementation based on five trials (Table 4) [70]. Four of the five studies tested recurrent NTD pregnancies, and one trial assessed first-time NTD occurrence. The meta-analysis weighted the MRC study 47.58% (Analysis 1.1 [70]). There were no protective or negative effects of folic acid supplementation on other congenital abnormalities or maternal outcomes. Of note, baseline NTD prevalences are divergent between the studies (Table 4).

The authors frequently conflate folate with folic acid, stating, “supplementing with folate” when the studies under analysis supplemented with folic acid or a multivitamin/mineral mix. Declared interests by authors include multiple organizations with investments in the micronutrient industry. The data in Table 4 is taken from the primary publications. Chi square statistical analysis of the individual studies demonstrates statistical significance with two of the five studies where a folic acid supplement significantly reduced recurrent NTDs in the MRC study [7] as well as first-time NTDs in the Czeizel study [34]. Regarding the Czeizel et al. study where participants from the Hungarian Family Planning Program trial begun in 1984 had no prior history of an NTD pregnancy, the baseline prevalence of NTDs was 29 per 10,000 births and was reduced to 0 per 10,000 births with folic acid supplements. This is a large difference in baseline NTDs compared to the Murphy and Westmark data for Hungary from 2013 at 10 NTDs per 10,000 births with no fortification, i.e., a 66% difference in NTD prevalence in the absence of folic acid food fortification comparing 1984 and 2013. Regarding the Kirke study [71], treatment groups were folic acid only, multivitamin excluding folic acid, and folic acid plus multivitamin. Inclusion criteria were women who had had a baby with an NTD and who were not pregnant but were planning a further pregnancy. The non-randomized, untreated cohort was 106 women who were pregnant at the start of the study and could not be randomized. The data was from informative pregnancies, and two comparisons were made, i.e., the proportion of births of NTDs occurring to women taking multivitamins (two-thirds of the samples) compared with the proportion occurring to women not taking multivitamin tablets (one-third of the sample) and the second comparison between women receiving versus not receiving folic acid. The study started as double-blinded, but blinding was partially lost during the study with a change in vitamin manufacturer and coloring.

Regarding the Laurence study [72], they parse their subjects based on good/fair/inadequate diet for folate and there was a 0% rate of recurrent NTDs in those with a good or fair diet (*N* = 50 out of 61 subjects in the control/untreated cohort) compared to six recurrent NTDs out of 11 subjects with an inadequate diet in the control group. Laurence et al. counted compliance at the 6th–9th week of estimated gestation if the serum folate concentration was higher than 10 mcg/L, and then the woman’s account of taking the tablets was considered valid. They include as non-compliers those with a serum folate concentration below 10 mcg/L as well as one woman with an exceptionally high serum folate of 212 mcg/L and normal RBC folate who did not take the folic acid tablets during early pregnancy, and took a large number of tablets at 7 weeks’ gestation, and her pregnancy was terminated at 3 months in a spontaneous abortion of an anencephalic fetus. The authors attribute trial failure to non-compliance with the supplement regimen. An alternative interpretation is that non-compliance should not be based on serum folic acid levels. (1) Details are not provided in the methods regarding the fasting status of women at the time of blood collection. Perhaps some samples were collected early in the morning after an overnight fast. While RBC folate levels are not meaningfully affected by recent food intake, serum folate/folic acid levels are significantly affected as a function of fasting [73]. (2) The authors measured both serum and RBC folate by a *Lactobacillus* microbiological assay, which measures total folate (folic acid and reduced folates). The assay works by growing bacteria in folate-free medium and measuring growth such that more growth is proportional to more folate. The assay is dependent on bioavailable folate. UMFA is not bioavailable until it is reduced to tetrahydrofolate forms, and different folate vitamers can produce different bacterial growth responses. It was not studied if the subjects had genetic polymorphisms such as *MTHFR* variants that affected folic acid conversion to folate. (3) The high folic acid exposure at 7 weeks’ gestation in one woman could have been toxic and contributed to the anencephaly/spontaneous abortion, thus raising concerns about a Goldilocks effect where too much or too little folic acid causes NTDs. Regardless of merging the compliant and non-compliant subjects, the small study size does not reach statistical significance.

Overall, the Cochrane meta-analysis was heavily weighted on the MRC study. The Czeizel (first-occurrence NTDs) and MRC (recurrent NTDs) studies indicate statistically significant reductions in NTDs in response to folic acid supplementation, but neither study population is representative of the United States, which used this data to support food fortification with folic acid. The baseline NTD prevalences are very high at 29 per 10,000 in the Czeizel study and 412 per 10,000 in the MRC study. Hungary has vastly different economic conditions than the United States. The MRC study population was strictly recurrent NTD pregnancies, which are likely disproportionately affected by factors other than folate deficiency alone. The only first-time occurrence NTD study tested folic acid in the presence of other micronutrients. Of importance, these studies were conducted prior to widespread introduction of genetically modified foods and a large increase in the use of glyphosate. Bacteria in the gut that can reduce folic acid to folate include *Bifidobacterium* and *Lactobacillus*, which are sensitive to glyphosate [74,75]. Folate bioavailability in response to consuming folic-acid-fortified foods before and during pregnancy in countries with exposure to glyphosate remains to be determined.

The second Cochrane systematic review covers fortification of wheat and maize flour with folic acid for population health outcomes [48]. As mentioned above, this 95-page systematic review evaluated the efficacy of folic acid fortification of wheat and maize flour on health outcomes in the overall population, concluding that there was only one non-RCT that supports the contention that fortification of wheat flour may reduce the risk of NTDs (spina bifida and encephalocele but not anencephaly) with “very low certainty” regarding the efficacy of folic acid fortification in reducing NTDs [48]. The authors acknowledge limitations including the small number of studies and participants, limitations in study design, and low certainty of evidence as well as no studies reported on the occurrence of adverse effects. They conclude that fortification of wheat or maize flour with folic acid alone or with other micronutrients may increase erythrocyte and serum/plasma folate concentrations.

**Figure 2:**
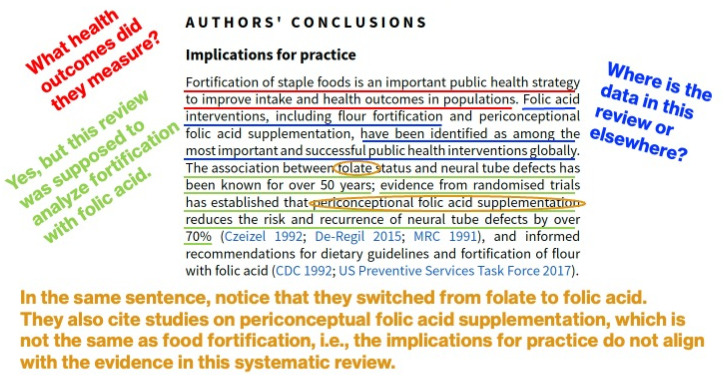
Authors’ conclusions and implications for practice from Cochrane systematic review [48] on fortification of wheat and maize flour with folic acid for population health outcomes.

Briefly, there were several design problems in the included studies, including dose, study timeframe, none of the RCT reporting data on NTDs, and lack of no intervention controls in the RCTs. Seven of the ten studies in the review were conducted in lower-middle-income or upper-middle-income countries including South Africa, China, Bangladesh and Mexico without consideration of how changing SES conditions affected outcomes. With no non-intervention controls, it is not possible to discern how increased access to food confounds the data. Within the report, the authors’ conclusions/implications for practice contradict the evidence (Figure 2), and many readers only read abstracts and conclusions, particularly for well-respected publications like Cochrane.

## 6. Need for Reevaluation of the Existing Literature

As described in the preceding section, recent systematic reviews rely on published data from decades ago without critical analysis of potential confounding issues and potential harms. A recent review article synthesized recommendations for folic acid supplementation after reviewing 10 clinical practice guidelines, four expert consensus statements, two recommended practice documents, and one best practice document, while excluding primary research and systematic reviews, and concluded: “For clinical practice, the evidence strongly supports a risk-stratified supplementation strategy: (1) all women of childbearing age should take 0.4 mg of folic acid daily starting at least 3 months before conception; (2) women at moderate risk require 1.0 mg daily; and (3) high-risk women require 4–5 mg daily. The findings affirm that dietary sources alone are insufficient to meet recommended levels for NTD prevention, and routine serum and metabolic testing for folate is not recommended for most women of childbearing age” [76]. While this is an accurate assessment of current expert opinion in the field, the recommendations are fundamentally flawed. Supplements may be indicated in specific populations or clinical contexts, but they are not universally required.

Dietary sources of folate alone can be sufficient for NTD prevention, i.e., the highest NTD prevalence at 32 per 10,000, or 0.32%, and no fortification with folic acid equates to 99.68% of pregnancies having no NTD. Conversely, considering a potentially high rate of supplementation at 30% of women of childbearing age in a population, diet is still sufficient to prevent NTDs in most pregnancies. For comparison, in the United States a rare disorder is defined as affecting fewer than 200,000 people, which equates to 6 per 10,000 and is higher than the prevalence of NTDs. In the case of rare disorders, the policy is to screen and treat individuals. It would be a more logically sound position to promote routine folate screening and metabolic testing in women of childbearing age to identify subpopulations that may require supplementation.

Reliance of current reviews on the same handful of studies that lack data on important confounding issues and harms is advancing a self-reinforcing loop that promotes folic acid food fortification despite limited supporting evidence. A one-size-fits-all approach to health ignores biological differences between individuals; diverse social, cultural and environmental contexts; unequal disease risk and burden; variation in treatment response and side effects; and ethical concerns while reducing the effectiveness of preventive strategies and impeding patient-centered care and scientific understanding of subgroup effects. Concurrently, there is substantial mis-citation of publications that do not support food fortification. Using Murphy and Westmark [46] as an example, bibliometric analysis indicates a 70% inaccuracy rate in publications citing the work, including 21 citations with incorrect/opposite finding errors [77]. Of concern, publications from countries with mandatory fortification of wheat had an increased number of serious citation errors, i.e., 64% of the papers in which the corresponding author was from a country with mandatory fortification had serious errors, whereas 77% of papers from authors in countries without mandatory fortification cited Murphy and Westmark correctly. The research process and public health policy are dependent on the accurate citation and critical evaluation of prior scientific research.

## 7. Personalized Medicine

### 7.1. The Goldilocks Effect

Proponents of national food fortification with folic acid commit an exception fallacy, i.e., data about individual cases is used to draw conclusions about a group of people [52]. Just because inadequate folate levels can cause NTDs does not mean that all pregnant women will benefit from synthetic folic acid supplementation or fortification, nor most of the general population. There is no national monitoring program for consumption levels of folic acid at the individual level in response to fortification with correlation to health effects. Even if surveillance data demonstrated improvements in NTD outcomes within the intended population following fortification, the policy would still raise ethical concerns due to adverse health outcomes in other subgroups in response to folic acid.

There is a Goldilocks effect regarding medications where there is an optimal, effective middle zone that exists between two extremes such that too little is not effective and too much is toxic. It is common in biology regarding vitamins, micronutrients and medications where more is not always better. It is impossible to optimize folate and folic acid consumption at the individual level with a one-size-fits-all national policy. The offspring of pregnant women consuming too little folate are at risk of NTDs. People consuming high levels of fortified foods can be in a state of folic acid excess. Living organisms are complex adaptive systems with nutrients acting in tightly interconnected networks. Fortification of cereal grains with folic acid in the United States exposes approximately 100,000 people to a synthetic vitamin for each case of NTDs potentially prevented and elicits the question of why a comprehensive pre-conception package is not considered as a viable precision medicine approach to prevent NTDs. A more targeted approach to improve the diet and SES of women of childbearing age would better serve everyone.

### 7.2. Reasons to Promote Personalized Medicine

There are many reasons to promote a personalized medicine approach to folate, including people responding differently to the same treatment, genetic and molecular diversity of disease, improved treatment effectiveness, reduced adverse drug reactions, earlier and more accurate diagnoses, better disease prevention, and cost efficiency. Regarding the differential response of people to the same treatment and genetic and molecular diversity of disease, much remains to be learned about what genes cause NTDs and how persons with certain genetic variants such as *MTHFR* differentially respond to folic acid. Half of the population is male, and folic acid is associated with prostate cancer [78]. Many women are not of childbearing age and excess folic acid is associated with masking B12 deficiency. Thus, it would be more equitable to treat individual patients to reduce NTDs instead of targeting the whole population.

In terms of improved treatment efficacy, current fortification policies are not reaching the entire targeted population [79] and likely causing adverse effects in the non-targeted population. For example, the NHANES 2001–2020 data indicated that 20% of girls aged 14–18 years had insufficient RBC folate [80]. At the same time, both males and females aged 1–3, 4–8 and 9–13 years had greater than 50 nmol/L serum folate, which is above the traditional upper limit for serum folate for children at 47 nmol/L. There is a lack of non-confounded data demonstrating that fortification with folic acid reduces NTDs. Strategies to improve overall nutrition, including a focus on folate-rich foods targeted to women of reproductive age, would reduce folate deficiency and associated NTDs. Adverse effects associated with high levels of folic acid include masking vitamin B12 deficiency leading to nervous system damage, anemia, cognitive impairment and neuropathy; UMFA in the bloodstream; promotion of existing tumor growth; altered neurodevelopment, epigenetic effects, and potential gene–nutrient interactions; and interference with chemotherapy and antiepileptic drugs [24,43,81,82,83,84,85,86,87,88,89,90,91]. We have much to learn regarding potential effects on epidemics of Alzheimer’s disease, autism and obesity as well as cancer and other diseases and conditions as a function of diet and genetics.

Using autism and *MTHFR* gene variants as an example, too much or too little folic acid is a risk factor for autism [92,93,94,95,96,97,98]. MTHFR is a critical enzyme in folate metabolism that affects DNA methylation, synthesis and repair. People with the *MTHFR* C677T polymorphism have problems converting folic acid to the active folate metabolite 5-MTHF (the form that is transported into cells), leading to increased homocysteine levels and a higher risk of miscarriages and NTD pregnancies [99,100,101]. The *MTHFR* C677T genetic variant is homozygous in approximately 12% of individuals in North America and heterozygous in 40–45% [102]. Thus, folic acid fortification may be ineffective, or worse, toxic, in persons with *MTHFR* C677T polymorphisms. In addition, UMFA has the potential to bind to folate receptors and block access of 5-MTHF from the diet [99]. And folate receptor alpha (FR-α) autoantibodies are associated with worse adaptive functioning scores on the Vineland Adaptive Behavior Scales (VABS) in children with autism [103]. The rates of autism have increased substantially since 1998 when folic acid fortification was implemented in the USA [75]. An increased probability of consuming folic acid from voluntary food fortification was associated with greater risk of intakes above the UL in children [104]. In the NHANES 2011–2012 study, the average serum total folate levels were 61.2 (1–5 years old) and 60.4 (6–11 years old) nmol/L [105]. Prior to fortification of food products with folic acid, the typical physiological range of folate was 11.3–47.6 nmol/L. In a study from Brazil post-folic-acid-fortification, over 10% of children age 12–19 years had plasma folate levels greater than the typical physiological range with males at 50.5 nmol/L and females at 54.6 nmol/L for the 90th percentile and males at 58.7 nmol/L and females at 67.6 nmol/L for the 95% percentile [106]. Serum UMFA is highest in individuals over 60 years of age and in children [73], i.e., age groups more vulnerable to Alzheimer’s disease and autism, respectively.

Personalized medicine will be required for earlier detection and prevention of NTD risk. Molecular biology tools have grown rapidly in the past three decades and unit costs have fallen substantially. Whole genome sequencing can detect rare disease mutations, cancer risk and pharmacogenomics. Epigenomic technologies like DNA methylation and chromatin profiling inform environmental exposure effects. Transcriptomics, proteomics and metabolomics can predict treatment response. Microbiome profiling can inform personalized dietary advice. We currently lack a good understanding of the interplay between multifactorial factors (nutrition, genetics, metabolism, environment, medications) in the development of NTDs. The strongest studied factor to date is folate insufficiency but other factors such as one-carbon metabolism gene variants, pre-gestational diabetes, obesity, medications including valproic acid, pesticides, hyperthermia in early pregnancy, infection, and pre-conception care also play a role. The integration of omics technologies and microbiome tools, in collaboration with improved artificial intelligence and machine learning, offers the promise of discerning the molecular mechanisms underlying failure of the neural tube to close and formulating preventive strategies with feasible clinical utility and cost effectiveness.

Overall, the evidence supporting folic acid food fortification for the prevention of NTDs is weak at best and, in the worst case, harmful to large subgroups of the population. There are numerous ethical considerations that support an individual-level versus a population-level approach to folate/folic acid exposure [107]. With national fortification, individuals cannot opt out, the benefits and risks are not evenly distributed, and there is a lack of transparency regarding potential harm.

### 7.3. Moving Foward

An important issue is how to move forward when fortification is so heavily entrenched in current policy. It would not be easy to un-mandate food fortification of cereal grains with folic acid in the United States. Folic acid fortification of cereal grains was established under 21 CFR 137.165(a) in the Code of Federal Regulations by the Federal Drug Administration (FDA) [1]. The FDA has authority to regulate nutrients by setting the standards for required and voluntary labeling of foods and supplements. The process to issue rules is called “notice and comment rulemaking” and is a three-step process. First, the FDA issues a proposed rule, also called a notice of proposed rulemaking (NPRM). The NPRM explains what the FDA intends to require or do as well as the scientific and policy reasons for the rule. Second, public comment from industry, consumers and health experts is requested regarding the NPRM. Third, the FDA responds to comments, and if it decides to proceed, issues a Final Rule that is published in the Federal Register under Title 21 of Code of Federal Regulations. Regarding § 137.165, flour is defined in section § 137.105 as, “Flour, white flour, wheat flour, plain flour, is the food prepared by grinding and bolting cleaned wheat, other than durum wheat and red durum wheat”. Of note, other flours such as rice, almond, coconut, oat and chickpea that are commonly used by persons with celiac disease and wheat allergies or intolerances are not included in the law. Thus, to change fortification rules at the federal level, the FDA would need to be convinced to issue an NPRM, collect public feedback, and issue a Final Rule.

To issue an NPRM, the FDA would likely require rigorous evidence of risk such as epidemiological data showing no significant prevention of NTDs, adverse effects regarding vitamin B12 deficiency or cancer, and a comprehensive regulatory impact analysis determining the costs and benefits in terms of health and the economy. For reasons previously described, including lack of data on prospective monitoring, control groups, dosage and long-term effects, generation of these data is not feasible [108]. Major constraints to a government-agency-led reversal of fortification policy include lack of support from industry groups and proponents of food fortification. The perceived efficacy of folic acid is engrained (pun intended) in national health policy in the United States. High resistance would be expected from public health, nutrition and maternal and child health experts who rely on expert consensus statements from the World Health Organization (WHO) and CDC. No country has ever reversed its mandatory folic acid fortification policy once implemented.

The health concerns associated with UMFA in the United States in the context of overnutrition are predicted for all industrialized, developed countries with mandatory folic acid fortification policies while different challenges are predicted with fortification in developing countries in the context of undernutrition. In both scenarios, too little folate and too much folic acid are predicted to be associated with adverse health outcomes.

Considering the extensive EBL accumulated in the past three decades, as well as the common sense approach to health and medicine of “first, do no harm”, both of which have not been taken into account by public health authorities in regard to folic acid, a grassroots approach to changing fortification policy will likely be more successful in the short-term than an FDA mandate. The inability to definitively prove adverse health effects associated with folic acid fortification could be equated with the state of affairs surrounding cigarette smoking in the 1950s when smoking was generally regarded as safe. The Surgeon General eventually warned of the health hazards of smoking despite the lack of any RCT that proved smoking caused lung cancer. Eventually evidence accumulated to the point where an RCT would be unethical. One could envision a similar scenario unfolding regarding food fortification with accumulating evidence of adverse health effects that could be parsed based on the default fortified American diet versus a control group who voluntarily avoid folic-acid-fortified foods and correlation with blood-based laboratory test results archived through national databases such as All of Us.

Consumer-led change is possible in the arena of food fortification. After public health agencies determined that sodium intake was a major risk factor for hypertension and cardiovascular disease, government agencies set voluntary targets for bread manufacturers while consumer advocacy groups campaigned for lower sodium in processed foods. The increased awareness and pressure for change pushed food manufacturing companies to reformulate products, resulting in a downward trend in sodium content in packaged foods. Although progress was slow, change could be made. Economists estimate that 10–20% of consumers changing their purchasing habits for staple goods can trigger production changes. Using gluten-free foods as an example, about 1% of the global population has diagnosed celiac disease requiring a strict gluten-free diet while 10.1% self-report non-celiac gluten/wheat sensitivity (NCGWS) with a female predominance [109]. In the United States, self-reported NCGWS is 5.1%. Of individuals with self-reported NCGWS, 40% adhere to a gluten-free diet. It is estimated that 31% of adults in the United States eat gluten-free or try to avoid gluten, which is well beyond the 4% that avoid gluten for medical reasons [110]. The gluten-free market has expanded from specialized dietary needs to a health movement with a projected compound annual growth rate (CAGR) of 8.33% [111]. North America dominants the market share at 36.7%. Hence, consumer demand for gluten-free products, predominantly fed by celebrity endorsements and emergence of gluten-free as a fad diet for weight loss, has significantly impacted food manufacturing.

There is no mandate for fortification of gluten-free flours. Persons eating gluten-free are not consuming folic-acid-fortified cereal grains, although these flours may be fortified with other nutrients such as vitamin D, calcium, potassium, thiamin, riboflavin and niacin, dependent on manufacturer. Hence, it is possible to avoid folic acid in the diet by adopting a gluten-free diet. It should be noted that there is a big difference between eating gluten-free and eating a healthy gluten-free diet. Processed gluten-free foods typically contain high fat and sugar. In the absence of eating fresh fruits and vegetables, there is a high propensity for vitamin deficiencies. The other downsides of eating gluten-free are the cost premiums, inconvenience of shopping at multiple grocery stores to find products, being selective about eating out, and planning when traveling. Overall, the healthiest strategy to avoid folic acid in the food chain is to adopt a healthy gluten-free diet that includes whole foods and an abundance of fresh fruits and vegetables. There are numerous online tools and apps that track macro- and micronutrients in the diet. It is possible to consume daily recommended allowances of micronutrients by eating whole, nutritious foods.

Further considerations that could impact consumer preferences include the environmental impact associated with producing synthetic folic acid. The main route of folic acid production is chemical synthesis, which requires condensation of a pteridine ring with para-aminobenzoic acid (PABA) and glutamic acid followed by oxidation and reduction steps to form the full pteroyl-glutamate structure. Purification involves crystallization, alkali dissolution, adsorbents and additional acid crystallization steps to achieve pharmaceutical and food grade purity. This multistep process is energy-intensive, contributing to greenhouse gas emissions, and it generates chemical waste. The transportation and packaging of folic acid for global distribution also contribute to carbon emissions. The global folic acid market was valued at USD 1.04 billion in 2024 and is projected to grow at a CAGR of 5.1% from 2025–2030 [112]. Approximately 160,000 metric tons of folic acid and folate derivatives are produced per year. Mandatory folic acid fortification programs in 90+ countries are the top driving force for production of synthetic folic acid. This is a large enterprise based on dubious data.

In summary, consumer preference and market trends are rapidly moving toward gluten-free food, which is also folic-acid-free. This grassroots change in dietary pattern and capitalism-based manufacturing practice will likely be more effective in reducing folic acid in the food supply than a public-health-agency-led initiative to reevaluate the EBL and come to a consensus to change mandatory fortification requirements in the context of strong opposition from food fortification proponents. With that said, consumers prefer to maintain the status quo [113], and change from the default is expected to be slow and biased. Access to healthy foods and nutrition education as early as possible are paramount [114]. Educational institutions and employers could facilitate setting smart defaults through changes in school meal programs and health insurance incentives. Education campaigns could be implemented for public messaging on folate requirements and sources. Nutrition education requirements could be implemented for high school, college and medical school programs. Social programs could facilitate acquisition of healthy foods and increased folate consumption in low-SES communities. Increased voluntary monitoring of food intake through phone-based apps and surveys for national surveillance could aid analysis of nutrient intake. A major problem with this slow grassroots change is that, with or without fortification, we will still have a substantial portion of women of childbearing age who are not meeting the RDA for folate consumption. There is some hope that the federal government could quickly mandate changes in food manufacturing as evidenced by the recent Make America Healthy Again (MAHA) movement to eliminate food dyes from processed foods, medications and products served in schools.

## 8. Conclusions

The strength of the EBL on folic acid fortification in reducing NTDs in the general population is limited while evidence is accumulating regarding ineffective coverage, overdosage, and probable adverse effects. Knowledge gaps include lack of prospective monitoring, control groups, dosage and long-term effect data. These voids are particularly relevant for individuals with gene variants that affect folate metabolism. It is past time to reassess mandatory national food fortification policies. Even though the health effects of folate are well-documented and supplementation with folic acid can be effective against recurrent NTD-affected pregnancies, a one-size-fits-all fortification approach to folate and folic acid consumption is subject to the Goldilocks effect and is not ethical.

## Figures and Tables

**Table 1:** Government and organization policies/recommendations on folic acid fortification.

Agency	Position
CDC	Recommends all women capable of becoming pregnant consume 400 mcg folic acid daily to prevent NTDs through supplements or fortified foods. It is also important to consume a varied diet with folate-rich foods.
FDA	Issued regulation §136.115 in 1996 requiring all enriched cereal grain products be fortified with folic acid by January of 1998 at levels ranging from 0.43 mg to 1.4 mg per pound.
Food Standards Australia New Zealand	Mandatory fortification of bread with folic acid is the preferred approach in Australia and New Zealand to further reduce the incidence of NTDs. The proposed level of mandatory fortification is 80–180 mcg of folic acid per 100 g of bread. (Equates to 363–816 mcg/pound.)
Global Alliance for Improved Nutrition	Folic acid fortification is an evidence-based intervention that reduces the prevalence of NTDs, and large-scale food fortification with folic acid is underutilized.
Health Canada	Canda mandates fortification of white flour with folic acid.
International Society for Pediatric Neurosurgery	Recommends that all governments require mandatory folic acid fortification of centrally produced food staples to provide almost all women of reproductive age who eat fortified foods with at least an additional 150 mcg/day of folic acid as recommended by the WHO.
Institute of Medicine (National Academy of Medicine)	All women capable of becoming pregnant should consume 400 mcg of synthetic folic acid daily from fortified foods or supplements in addition to a healthy diet.
Public Health Service	Recognized the link between folic acid intake and NTDs as a compelling health issue in 1992 and recommended that all women of childbearing age should have adequate folate intakes (0.4 mg daily) throughout their childbearing years, but warned that the total intakes should not exceed 1 mg.
UNICEF	UNICEF states they improve the quality of women’s diets through large-scale food fortification programs like salt iodization and the fortification of white flour, rice, and cooking oil with vitamins and nutrients.
United Kingdom Food Standards Agency	Government advice for women who are pregnant or trying to conceive is that they consume a folic acid supplement of 400 mcg per day ideally 3 months before and for at least the first 12 weeks of pregnancy. Under new requirements, non-wholemeal wheat flour will be fortified with folic acid by law from December 2026. Fortification is intended to support, not replace, current advice on folic acid supplementation. Individuals who cannot or choose not to eat fortified products can choose products made from wholemeal flour, gluten-free products and other types of four such as soya or ancient grains like spelt.
United States Agency for International Development (USAID)	Strongly supports folic acid interventions primarily through large-scale food fortification and maternal supplementation in alignment with WHO and U.S. Public Health Service recommendations with an emphasis on population-level delivery over individual supplementation alone.
U.S. Preventive Services Task Force (USPSTF)	Recommends that women planning to or who could become pregnant take a daily supplement containing 0.4 to 0.8 mg of folic acid.
WHO	Recommends daily folic acid supplementation with 400 mcg folic acid for pregnant women ideally commencing before conception and until 12 weeks’ gestation to prevent NTDs.
World Bank/Ministry of Public Health	Recommends 400–800 mcg folic acid per day for antenatal care.

**Table 2:** NTD prevalence per 10,000 as a function of SES (data reproduced from Murphy and Westmark [46]).

SES Quintile	With Fortification	No Fortification
1 (highest)	8.90	11.74
2	10.87	12.45
3	13.11	10.50
4	15.38	16.19
5	16.90	15.82

**Table 3:** Moges NTD data merged with World Bank Group GDP data.

Study	Country	Time Period	NTDs per 10,000 Births	GDP in Billions of US Dollars
1. Amarin	Jordan	2000–2001 (pre-fortification)	18.5	8.46–8.98
		2002–2004 (transition)	10.7	9.58–11.41
		2005–2006 (post-fortification)	9.5	12.59–15.06
2. Barboza-Arguello	Costa Rica	1987–1991 (pre)		4.53–7.22
		1996–1998 (pre)	9.8	11.68–13.68
		2003–2012 (post)	4.8	17.27–47.23
3. Castilla	Chile	1999 (pre)	26	75.58
		2000 (transition)	21	78.34
		2001 (post)	16	71.57
4. Cortes	Chile	1999–2000 (pre)	17.1	75.58–78.34
		2001–2009 (post)	8.6	71.57–171.78
5. Godwin	Canada	1992–1996 (pre)	4.9 (spina bifida)	594.39–630.61
		1999–2003 (post)	2.5	678.41–895.54
6. Hilder	Australia	Oct 2006-Dec 2008 (pre)	12.8	749.71–1.06 × 10^3^
		Jan-Sept 2009 (transition)	11.8	931.76
		Oct 2009–Mar 2011 (post)	11.2 (not significant)	931.76–1.4 × 10^3^
7. Liu	Canada (Newfoundland)	1991–1997 (pre)	43.6	deep recession early 1990s
		1998–2001 (post)	9.6	new industries late 1990s
8. Lopez-Camelo	Chile	1982–1989 (pre)	9.31 (spina bifida)	19.71–30.1
		1990–2000 (pre)	9.32	33.43–78.34
		2001–2002 (post)	4.77	71.57–70.26
9. Persad	Canada	1991–1997 (pre)	25.8	deep recession early 1990s
	(Nova Scotia)	1998–2000 (post)	11.7	improved economy late 1990s
10. Poletta	Chile Argentina	1990–2000 (pre) 2001–2013 (post) 1990–2004 (pre) 2005–2013 (post)	10.85 males 18.90 females 5.54 males 5.55 females 10.20 males 18.59 females 7.25 males 7.56 females	33.43–78.34 71.57–277.4 141.35–164.66 198.74–552.03
11. Ray	Canada	Jan 1994–Dec 1997 (pre)	11.3	recession early 1990s; NAFTA 1994; Common Sense Revolution 1995–2002
	(Ontario)	Jan 1998–May 2000 (post)	5.8	
12. Santos	Brazil	2001–2004 (pre)	7.9	559.58–669.29
		2005–2014 (post)	5.5	891.63–2.46 × 10^3^
13. Tarqui-Mamani	Peru	2001–2005 (pre)	12.1	52.03–76.05
		2006–2010 (post)	10.1	88.64–147.53
14. Williams	United States	1995–1996 (pre)	6.5	7.64 × 10^3^–8.07 × 10^3^
		1999–2011 (post)	4.0	9.63 × 10^3^–15.6 × 10^3^

**Table 4:** Summary of studies included in the 2015 Cochrane review on folic acid supplementation and NTDs.

Citation	Year	Country	Controls	Treated	Chi Square, *p*
Czeizel	1992	Hungary (no prior history NTD pregnancy) -folic acid plus other micronutrients	6/2052, 0.29%	0/2104, 0%	0.013
ICMR	2000	India (prior history NTD pregnancy) -folic acid plus other micronutrients	10/142, 7.0%	4/137, 2.9%	0.11
Kirke	1992	Ireland (prior history NTD pregnancy)	3/103, 2.9%		
		-multivitamin without folic acid		1/89, 1.1%	0.39
		-folic acid		1/85, 1.2%	0.41
		-folic acid plus multivitamin		2/87, 2.3%	0.79
Laurence	1981	Wales (prior history NTD pregnancy)	4/51, 7.8%	2/60, 3.3%	0.30
		-folic acid compliant		0/44, 0%	0.058
		-non-compliant		2/16, 12.5%	0.57
MRC	1991	Australia, Canada, France, Hungary, Israel,	10/243, 4.1%		
		Russia, UK (prior history NTD pregnancy)			
		-control: Fe sulfate and Ca phosphate			
		-folic acid		1/242, 0.41%	0.0062
		-vitamin mix without folic acid		7/234, 3.0%	0.51
		-folic acid plus vitamin mix		2/241, 0.83%	0.020

## Data Availability

Data is contained within the article.

## Abbreviations

The following abbreviations are used in this manuscript:

CAGR	Compound annual growth rate
CDC	Centers for Disease Control and Prevention
DFE	Dietary folate equivalents
DHF	Dihydrofolate
DHFR	Dihydrofolate reductase
EBL	Evidence-based literature
FFI	Food fortification initiative
GDP	Gross domestic product
Hcy	Homocysteine
Met	Methionine
MRC	Medical Research Council
5-MTHF	5-methyltetrahydrofolate
5,10-MTHF	5,10-methylenetetrahydrofolate
MTHFR	MTHF reductase
NAMS	National Academy of Medical Sciences
NCGWS	Non-celiac gluten/wheat sensitivity
NPRM	Notice of proposed rulemaking
NTDs	Neural tube defects
PABA	Para-aminobenzoic acid
RCT	Randomized clinical trial
RBC	Red blood cell
SAH	S-adenosylhomocysteine
SAM	S-adenyl-methionine
SES	Socioeconomic status
THF	Tetrahydrofolate
UL	Upper tolerable limit
UMFA	Unmetabolized folic acid
USD	United States dollars
WHO	World Health Organization
